# Seeking Health Care for Perimenopausal Symptoms: Observations from The Women Living Better Survey

**DOI:** 10.1089/jwh.2022.0230

**Published:** 2023-04-04

**Authors:** Marcie K. Richardson, Nina Coslov, Nancy Fugate Woods

**Affiliations:** ^1^ Department of Obstetrics and Gynecology, Atrius Health and Beth Israel Deaconess Medical Center, Boston, Massachusetts, USA.; ^2^ Women Living Better, Cambridge, Massachusetts, USA.; ^3^ Department of Biobehavioral Nursing and Health Informatics, University of Washington, Seattle, Washington, USA.

**Keywords:** perimenopausal health care, Midlife Women's Health Care, menopause, anticipatory guidance, perimenopausal health care interactions, health care satisfaction

## Abstract

**Background:**

The perimenopausal health care interaction can be challenging both for those seeking care and health care professionals (HCPs). We explore the factors that contribute to making these health care interactions either satisfying or unsatisfying for a midlife person with ovaries who consults an HCP about bothersome symptoms.

**Materials and Methods:**

Respondents to the Women Living Better (WLB) survey were asked about 61 symptoms often associated with the menopausal transition. They were then asked whether they sought health care for their most bothersome one. Of the 1024 participants who consulted an HCP, 964 provided a response to the open-ended question “how did that go?” We used conventional content analysis to code the responses and identify themes.

**Results:**

We identified six codes reflecting positive health care interactions which we then grouped into five themes suggesting satisfaction with these health care interactions. These included: validating experiences; having matching explanatory models; being supported by a team; engaging in shared decision-making; and having symptoms addressed. We identified 13 codes reflecting negative health care interactions which we then group into 4 themes suggesting dissatisfaction. These included: invalidating experiences, a mismatch in expectations between care recipients and HCP, barriers to treatment, and not feeling helped.

**Conclusions:**

Those seeking health care for bothersome symptoms on the path to menopause responded with both positive and negative comments about health care interactions in the WLB survey. Studying these comments identifies opportunities to improve midlife care.

## Introduction

Health care interactions are complex exchanges which can result in either satisfied or dissatisfied care recipients. Duggan and Street offered a framework to analyze health care professionals' (HCPs) efforts to combine task-oriented and relational functions during the health care interaction.^1^ They describe task-driven functions which include exchanging information, enabling self-management, supporting shared decision-making, and managing uncertainty; and relational functions which include fostering healing relationships and validating and responding to a patient's emotions, experience, and personal context. On the part of those seeking care, positive task-related outcomes are identified as embracing HCP treatment plans, adhering to treatment, and feeling satisfied with decisions. Positive relational-related patient outcomes include feeling understood, “known,” involved in their care, and experiencing rapport with and trust in their clinicians. Greene and Ramos report actively listening, providing detailed explanations, showing care for patients, and demonstrating their knowledge as fundamental to building trust with health care recipients (HCRs).^2^

In the Melbourne Women's Midlife Health Project, Dennerstein and colleagues documented that 30% of their participants who were at baseline 45–55 years old and still menstruating consulted an HCP annually while those with symptoms attributed to the menopause transition did so more often.^3^ These health care interactions at midlife when those seeking care are often perimenopausal present particular challenges. The path to menopause, a natural phenomenon, is unique for each individual, and symptoms often begin before they are anticipated.^4^ In addition, many HCPs report not being adequately trained to address perimenopause related issues.^5^

Meanwhile, perimenopause has been getting more and more attention in mainstream media.^6–9^ This coverage reveals knowledge gaps and controversies around perimenopausal physiology and best practices to manage its manifestations and health consequences. HCP and people seeking care can have discordant expectations, goals, and explanatory models for symptoms experienced. A recent survey from the United Kingdom revealed a knowledge gap for both HCRs and those seeking care and noted that perimenopause was often a hidden phenomenon. This same survey demonstrated differing schools of thought among HCPs regarding managing symptoms as a theme expressed by perimenopausal HCRs.^10^ All of the above can contribute to making it difficult for the HCP to deliver satisfying care to those on the path to menopause.

To date most study of the health care interaction on the path to menopause has been focused on decision-making about hormone therapy.^11–13^ Greater research attention to health care interactions that take on the broader task of helping people with ovaries anticipate and navigate the physical and psychological effects of this life transition is needed. Such research will enable HCPs to improve perimenopausal health care interactions.

The purposes of the analyses presented here are to: (1) characterize the elements of a health care interaction about a bothersome midlife symptom that led to satisfaction or dissatisfaction on the part of the person seeking care and (2) offer ideas and strategies that might improve perimenopausal health care interactions.

## Materials and Methods

### Design of sampling strategy

Our data come from responses to an online questionnaire, the Women Living Better (WLB) survey, hosted on SurveyMonkey which was open from March to August 2020. A convenience sample of participants aged 35–55 was recruited from the WLB website, newsletter, and by e-mail among online networks with links posted on social media channels of WLB and other groups. Methods are described in detail elsewhere.^4^ The University of Washington Office for Human Subjects Division determined that the survey was exempt from further human subjects' review.

### Measures

The WLB survey included 82 items. We asked about current menstrual patterns. Then participants were queried about 61 symptoms possibly related to the hormone shifts of perimenopause, their frequency, and degree of bother. Respondents were then asked if they sought care for their most bothersome symptom. Those who answered “yes” were invited to respond to the question “How did that go”? The analysis here is based on text responses to that open-ended question.

### Data analysis

We used a qualitative method, specifically a conventional content analysis, as described by Hseih and Shannon^14^ to identify themes in the responses. We used an open coding approach to analyze the text of responses to generate codes for each response. Once we had a list of codes, we grouped related codes into themes.

**FIG. 1.**
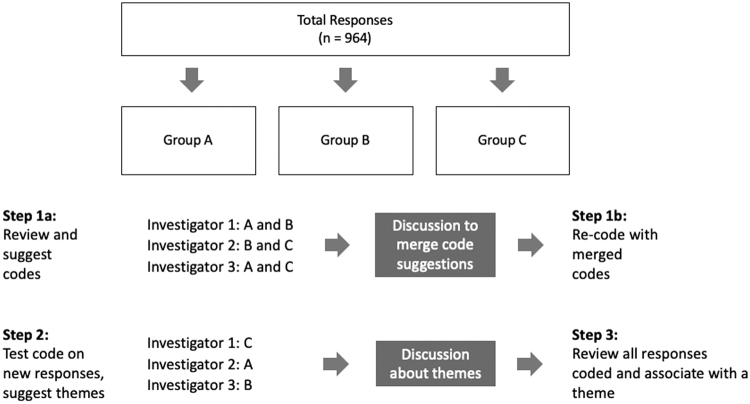
Steps in coding process and theme generation.

Specifically, and as shown in Figure 1, there were 964 responses which we divided into three equal groups for analysis. In step 1, each of the three investigators reviewed two of the three groups to analyze text and suggest codes reflecting participants' report of their experiences. After iterative reviews and discussion, consensus was reached on the final coding schema. It included 20 descriptive codes; 6 were associated with positive comments; 13 with negative comments; and 1 code identified comments that were neutral or had insufficient information to evaluate.

In step 2, each investigator analyzed the third set of responses which they had not previously seen to determine that the coding schema worked and whether saturation in codes was reached. We also identified overarching themes across the codes.

In the final step, we reached consensus on grouping codes and their alignment with themes. The six codes representing a positive interaction were grouped into five themes that reflected satisfaction with the health care interaction (see Table 1). The 13 codes representing a negative interaction were grouped into 4 themes associated with dissatisfaction (see Table 2).

**Table 1.** Themes, Codes, Definitions, and Illustrative Quotations Associated with Satisfying Health Care Interactions

Themes	Code	Definitions • Illustrative Quotations
S1. Validating Experiences	S1.1 Heard and Supported	S1.1 Uses the phrases “I was listened to,” “felt helped,” “my provider supported me.” • “Very sympathetic and helpful. Looked at all lifestyle elements and put me on anti-depressants and a plan to make other life changes.” • “It went well. My doctor made me feel heard and supported.”
	S1.2 Normalized or Acknowledged Link to Perimenopause or Age	S1.2 Use of word “normal” related to my age, stage of life, this phase, or to peri/menopause • “My doctor assured me it is normal.” • “New PCP compassionate and went out of her way to normalize it.” • “Doc said it sounds like menopause, it can take a long time and it's different for every woman.”
S2. Matching Explanatory Models	S2.1 Matching Explanatory Models	S2.1 Illustration of a shared view between provider and patient. • “She said that it could be perimenopause. I felt she understood where I was at and let me know other patients of hers are going thru the same things. So I wasn't alone.”
S3. Supported by a Team	S3.1 Supported by Team	S3.1 Mention of a team or more than one provider • “I've been working with a whole team of healthcare providers over the last several years, I love them all and couldn't get by without them.” • “In discussing symptoms with both by gynecologist and PCP, we decided to address my anxiety with exercise, meditation and diet, and will revisit w/both providers at my annual checkups.”
S4. Shared Decision-Making	S4.1 Shared Decision-Making	S4.1 Use of the word “we” • “I have a great health care provider who is very supportive, and we continue to work through all the symptoms.” • “My PCP recommended anti-depressants. I recommended St. John's Wort. She agreed with the St. John's Wart, but said if that did not work, we should revisit the antidepressants.”
S5. Symptom Addressed	S5.1 Symptom Addressed	S5.1 Patient notes that symptom was addressed with positive affect • “They put me on a small dose of Prozac and I am doing much, much better!” • “Good. Doctor said it was normal and gave me Xanax which I take only if I am having a full-on panic attack.”

**Table 2.** Themes, Codes, Definitions, and Illustrative Quotations Associated with Dissatisfying Health Care Interactions

Themes	Code	Definitions • Illustrative Quotations
D1. Invalidating Experiences	D1.1 Dismissed	D1.1 Uses the word “dismissed” or “brushed off” or “didn't listen” • “Initially, I was completely dismissed when discussing all of my perimenopause symptoms. Two doctors dismissed me, one quite rudely. I now work with a private hormone specialist and am getting the help I need.” • “I was brushed off, told that I am getting older.”
	D1.2 Too Young, Regular Period	D1.2 Mention of “too young” or “regular periods”• “It's not hormonal. You're too young and healthy and have normal periods. It has been beyond frustrating.” • “Consulted my OB/GYN about perimenopause and mood swings, sexual interest. She blew me off and said I was too young for menopause.”
	D1.3 Acknowledge Perimenopause, but it is “just how it is”	D1.3 Phrases like “It's just how it is” or “part of menopause” • “Doctor's response was welcome to perimenopause.” • “Welcome to your new normal.” • “I was told it was part of life.”
D2. Mismatch in Expectations	D2.1 Discordance Between HCR and HCP Views	D2.1 Alternate explanation offered by provider (busy life, depression) • “It didn't go well. they told me it was because I had teenage kids and a career and not because of my hormones even though I'm positive that's the reason.” • “She was compassionate‚ but the solution is always medication and that's not the route I want to go.” • “They want to throw sleep aids at it and that is not how I want to resolve this issue.”
	D2.2 Seeks Root Cause or Logic of Therapy	D2.2a Just offered a therapy but not a reason • “Not great. The migraines are a new symptom. I have only had them a few times, but they were debilitating. My doctor suggests I take Tylenol and lie down. I would prefer to address the cause and not just the symptom.”
		D2.2b Offered alternate therapy that isn't logical to patient (*e.g.*, SSRI for hot flashes) • “They wanted to prescribe an anti-depressant that I refused knowing it was hormonal.” • “Given antidepressant even though I told them I wasn't anxious or depressed.”
	D2.3 Perceived Incorrect Information from HCP	D2.3 HCP percieved as incorrect • “Dr. said it wasn't perimenopause if I wasn't having hot flashes.” • “Dr. wanted me on bcp- which I can't tolerate any more due to side effects.”
	D2.4 Conflicting Information from HCPs	D2.4 Hearing different things from different providers • “1st doctor said it was stress related/pre-burnout, second doctor prescribed birth control pills.”
D3. Barriers to Treatment	D3.1 Needed to See Multiple Providers	D3.1 Mention of more than one HCP; phrases such as: “I needed to see an X and a Y,” at first was dismissed but then saw a X. • “I had to go to an ob-gyn and 3 cardiologists before I found one who believed me and had knowledge that it could be linked to hormonal changes.” • “Not great. I saw three different GPs, was prescribed anti-depressants even though I said I thought it might be perimenopause, but I was told this couldn't be true as I still have regular periods.”
	D3.2 Expense of Treatment	D3.2 Cost mentioned • “Mental Health/therapy is $1500+ out of pocket. So, I declined.” • “Need surgery. Have no insurance.” • “She suggested pellets that aren't covered by my insurance that I can't afford.”
D4. Not Feeling Helped	D4.1 Given up	D4.1 Uses phrases such as: “I've given up” or “nothing to be done.” • “It seems there is nothing they can do.” • “No change. They just said it happens. Unsatisfying.” • “Insomnia is very hard to treat. I feel like I have given up.”
	D4.2 Testing done, but not helpful	D4.2 Uses phrases “No results from testing; testing was normal” • “Tests were run and they all came normal.” • “I was sent for a full blood screening and thyroid testing. All tests came back with good results, so my complaints were not addressed further.”
	D4.3 Received No helpful advice	D4.3 Uses phrases “Nothing helpful” • “Meh. I didn't feel helped.” • “Didn't get any real answers.” • “Still suffering.” • “They were not very helpful in giving me ways to address the issue.” • “Unsatisfactory” (several of this response)
	D4.4 Treatment Offered but Didn't Work	D4.4 Uses phrases “Treatment didn't work” • “Tried a sleeping pill which made me too groggy to function the next day. I would say - not well!” • They put me on weight loss medication that made me feel crazy. I had to stop taking it.” • “My doctor suggesting changing my medication for depression, but that did not go well.” • “I went on hormones. I was not able to sleep and was ready to kill someone.”

## Results

### Participants

Characteristics of the participants in the WLB survey are described in detail elsewhere.^4^ In brief, we utilized a convenience sample. Respondents were presumed female and ranged in age from 35 to 55 with a median age of 47 years. They were well educated; 81% had a college degree or more. A majority was employed at least part-time (87%), had daily responsibility for children or other dependents (67%), and were in a committed relationship (85%). Only 19% had difficulty paying for basic items. With respect to race/ethnicity: 85% identified as White, 6% as Hispanic/Latina, 2% as Asian, 3% as Black/African, 1% American Indian/Alaskan Native, 1% another race, and 2% preferred not to answer.

**FIG. 2.**
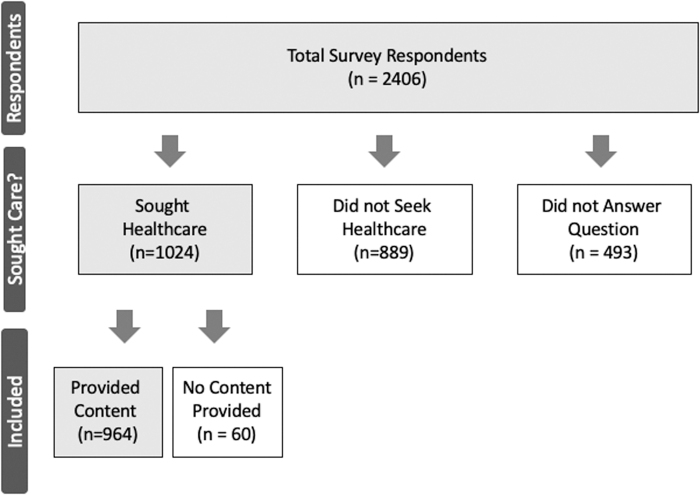
Yield of respondents included in content analysis.

As shown in Figure 2, of the 2406 participants who started the survey, 1024 endorsed consulting an HCP for their most bothersome symptom, 889 said they did not consult an HCP, and 493 did not respond. Of the 1024 who answered “Yes,” 964 provided a response to the open-ended question “how did that go?” Of those responding, 49% shared that experiences were interpreted as negative or dissatisfying; 18% reported experiences we interpreted as positive or satisfying. Nearly one-third of the responses (32%) were judged not to include enough content to definitively code. These included responses such as “Well I was prescribed an antidepressant” or “I was told it was the pre-menopause” or “started on birth control pills” answer without a clear positive (satisfied) or negative (dissatisfied) tone.

### Satisfying health care interactions

Themes that reflected satisfying interactions were as follows: validating experiences, matching explanatory models (*i.e.*, HCP understanding the person receiving care's perspective), being supported by a team, shared decision-making, and having symptoms addressed. Each theme is described below, and sample quotes are provided. Table 1 includes the themes, subthemes where applicable, codes, definitions, and illustrative quotations.

### Validating experiences

Validating experiences reflected empathy and support, reassurance that symptom(s) were “normal” or the affirmation that symptoms were typical for the care recipient's age and their progress on the path to menopause. We designated two subcategories:

**Heard and Supported.** Responses here used phrases such as “I was listened to,” “felt helped,” “my provider supported me.” An example, “[My HCP was] very sympathetic and helpful. Looked at all lifestyle elements and put me on anti-depressants and a plan to make other life changes.” Another, “It went well. My doctor made me feel heard and supported.”

**Normalized or Acknowledged Link to Perimenopause or Age.** Responses here included use of the word “normal” or “related to my age, stage of life,” “this phase, or to peri/menopause.” Examples: “My doctor assured me it is normal…” and, “New PCP compassionate and went out of her way to normalize it,”

“Doc said it sounds like menopause, it can take a long time and it's different for every woman.”

### Matching explanatory models

These responses indicated a shared view between the HCP and the person seeking care of the cause of symptom(s) and rationale for treating them. An example: “she said that it could be perimenopause. I felt she understood where I was at and let me know other patients of hers are going thru the same things. So I wasn't alone.”

### Supported by a team

This set of responses reflects a positive experience when multiple HCPs were involved in care. One example, “I've been working with a whole team of health care providers over the last several years, I love them all and couldn't get by without them.” Another, “In discussing symptoms with both my gynecologist and PCP, we decided to address my anxiety with exercise, meditation and diet, and will revisit with both providers at my annual checkups.”

### Shared decision-making

Responses in this group showed evidence of the person seeking care engaging in shared treatment planning or decision-making with their HCP. The use of the pronoun “we” was noted as participants described their experiences. Examples: “I have a great health care provider who is very supportive, and we continue to work through all the symptoms,” and “My PCP recommended anti-depressants. I recommended St. John's Wart. She agreed with the St. John's Wort, but said if that did not work, we should revisit the antidepressants.”

### Symptom addressed

The final category of responses associated with satisfaction was in instances where the symptom being addressed was coupled with a mention of improvement or resolution. An example, “They put me on a small dose of Prozac, and I am doing much, much better!” Another, “Good. Doctor said it was normal and gave me Xanax which I take only if I am having a full-on panic attack.”

### Dissatisfying health care interactions

Themes reflecting dissatisfying encounters included the following: invalidating experiences, a mismatch in expectations, barriers to treatment, and not feeling helped. Table 2 includes the themes, subcategories where applicable, codes, definitions, and illustrative quotations.

### Invalidating experiences

The responses coded as “invalidating experiences” had three subcategories: those having their concerns dismissed, those being told they couldn't be having symptoms related to perimenopause because of age or absence of cycle irregularity, or those being told “that's just how it is.”

**Dismissed.** Words used in this subcategory were “dismissed,” brushed off,” or “didn't listen.” Examples: “Initially, I was completely dismissed when discussing all of my perimenopause symptoms. Two doctors dismissed me, one quite rudely. I now work with a private hormone specialist and am getting the help I need,” and “I was brushed off, told that I am getting older.”

**Too Young or Regular Periods.** Expressions used here included either “too young” or “regular periods.” As an example, “It's not hormonal. You're too young and healthy and have normal periods. It has been beyond frustrating.” Another, “consulted my OB/GYN about perimenopause and mood swings, sexual interest. She blew me off and said I was too young for menopause.”

**Acknowledge Perimenopause, But It's Just How It Is.** Here phrases used included, “it's just how it is” or “part of menopause.” One participant shared, “Doctor's response was ‘welcome to perimenopause’.” Another was told, “Welcome to your new normal” and others, “I was told it was part of life.”

### A mismatch in expectations

Four subcategories represented a mismatch in HCP and the person seeking care's expectations: (1) discordance between expectations or explanatory model of the person seeking care's experience; (2) seeking but not receiving an explanation of the root cause of symptoms; (3) receiving what they perceived to be incorrect information about a remedy offered; or (4) receiving conflicting information from different HCPs.

**Discordance Between HCR and HCP Views.** Responses were coded as a “mismatch in explanation or approach” when the HCP offered an alternative explanation to perimenopause (*e.g.,* busy life, depression) or when the person seeking care wasn't interested in the therapy proposed. For example, “it didn't go well.they told me it was because I had teenage kids and a career and not because of my hormones even though I'm positive that's the reason.” Another was, “She was compassionate‚ but the solution is always medication and that's not the route I want to go.” Similarly, “They want to throw sleep aids at it and that is not how I want to resolve this issue.”

**Seeks Root Cause or Logic of Therapy.** Here responses noted a therapy offered without justification or one that didn't seem logical to the person seeking care (*e.g.,* SSRI for hot flashes). An example, “Not great. The migraines are a new symptom. I have only had them a few times, but they were debilitating. My doctor suggests I take Tylenol and lie down. I would prefer to address the cause and not just the symptom.” Another shared, “They wanted to prescribe an antidepressant that I refused knowing it (the symptom) was hormonal.” Another similar response, “[I was] given antidepressant even though I told them I wasn't anxious or depressed.”

**Perceived Incorrect Information from HCP.** This category reflected the person seeking care being offered information they believed was incorrect and/or offered a therapy they believed they should not use. For example, “Dr. said it wasn't perimenopause if I wasn't having hot flashes.” Another participant shared, “Dr. wanted me on [birth control pills] - which I can't tolerate any more due to side effects.”

**Conflicting Information from HCPs.** The fourth subcategory related to conflicting information among HCPs. As an example, “1st doctor said it was stress related/pre-burnout, second doctor prescribed birth control pills.”

### Barriers to treatment

There were two subcategories in the barriers to treatment theme: the need to see multiple HCPs and treatment perceived as too expensive.

**Need to See More than One HCP.** These responses suggested frustration with having to see more than one HCP. For example, “I had to go to an ob-gyn and 3 cardiologists before I found one who believed me and had knowledge that it could be linked to hormonal changes.” Others, “Not great. I saw three different GPs, was prescribed antidepressants even though I said I thought it might be perimenopause, but I was told this couldn't be true as I still have regular periods.” In addition to the need to see multiple HCPs leading to dissatisfaction, this example also reflects other codes such as a mismatch in explanatory models, dismissed as too young because of regular periods, and not feeling helped.

**Expense of Treatment.** Cost was another subcategory of barriers to treatment. One participant shared, “Mental Health/therapy is $1500+ out of pocket. So, I declined.” Another example, “Need surgery. Have no insurance.” And another, “She suggested pellets that aren't covered by my insurance that I can't afford.”

### Not feeling helped

The “not feeling helped” theme had four subcategories, including giving up, nothing to be done, testing done but not helpful, received no helpful advice, or offered a treatment that was ineffective.

**Given Up.** We coded responses as “given up, nothing to be done” when those precise phrases were used. Examples: “It seems there is nothing they can do,” “No change. They just said it happens. Unsatisfying,” and “insomnia is very hard to treat. I feel like I have given up.”

**Testing Done, But Not Helpful.** This subcategory included phrases like “no results from testing; testing was normal.” Examples: “tests were run, and they all came normal,” and “I was sent for a full blood screening and thyroid testing. All tests came back with good results, so my complaints were not addressed further.”

**Received No Helpful Advice.** Phrases here included “nothing was helpful” or “I wasn't helped.” Examples: “Meh. I didn't feel helped,” “Didn't get any real answers,” “still suffering,” “They were not very helpful in giving me ways to address the issue,” and several responses of “unsatisfactory.”

**Treatment Offered but Didn't Work.** This subcategory reflected interactions in which the HCR perceived the treatment offered to be ineffective. Examples: “Tried a sleeping pill which made me too groggy to function the next day. I would say - not well!,” “They put me on weight loss medication that made me feel crazy. I had to stop taking it,” “My doctor suggested changing my medication for depression, but that did not go well,” “I went on hormones. I was not able to sleep and was ready to kill someone.”

## Discussion

All people with a uterus and ovaries who live long enough will experience the menopausal transition. Previous research has shown that most are uncertain about what to expect^15^ and when to expect it.^4^ Some will arrive at menopause uneventfully, but others will encounter bothersome or even frightening symptoms that interfere significantly with their quality of life.^16–21^ Many will seek help from our health care system. The WLB survey responses illustrate a wide range of interactions between a person seeking care and their HCP during this stage of life. After establishing they had consulted an HCP about their most bothersome symptom, respondents were asked, “how did that go?” An analysis of comments to this open-ended question reveals insights that could improve midlife care, as well as suggesting health care system enhancements.

### Positive codes and satisfaction with heath care interactions

Our analysis identified several aspects of health care interactions that led to satisfaction. Interactions in which HCPs validated the person's experiences left them feeling heard and supported and as a result satisfied. Validation has many facets, including being present with the patient, making an accurate reflection of their expressions, restating their perspectives to ensure HCP understanding is accurate, normalizing the situation, and matching feelings as appropriate.^22–24^ Participants were pleased to be reassured that their symptoms were typical for their age or perimenopause. Based on a survey through her international newsletter and 13 years of receiving letters from those in perimenopause, Janine O'Leary Cobb, summarized, “women want to know that their menopausal complaints are fairly common.”^25^

The positive effect of validation has been explored in other health care settings such as communicating about pain. Some research suggests that HCP accepting that pain symptoms are understandable and legitimate can reduce negative effect and psychological distress for those seeking care.^26^ Epstein et al. concluded that quality of care improved when clinicians explored and validated patients' concerns in relation to prescribing for depression, concluding that participatory patient communication behaviors are more likely to be beneficial “when the physician meets that activated behavior with inquiry, openness, empathy, and validation, rather than cutoffs and assertion of his or her agenda.”^27^

Congruent explanatory models also led to satisfaction in our survey. Duggan and Street described relationship-centered care which “assumes that the interaction is co-created, such that the physician comes to a shared understanding of the patient's narrative through negotiated dialogue with the patient.”^1^ But, as we discuss below, validating symptoms as normal for stage or age and having a shared explanatory model may be challenging if the HCP hasn't received adequate menopause-related training and is unaware of wide ranging manifestations of the menopausal transition.

WLB participants indicated shared decision-making using “we” versus “my HCP” in their responses, which was interpreted as positive. Moreover, some participants also specified the value of being supported by a team of health professionals. In the editorial, “Who Cares for Midlife Women?,” Woods et al. noted a team approach as ideal in providing the broad care required during the menopausal transition.^28^

The theme focused on having symptoms addressed included statements about how symptoms were treated and participants' statements about whether their symptoms improved or resolved. It is not surprising that a successful therapeutic intervention, when it is possible, addressing the symptoms that the person seeking care prioritizes, results in an expression of satisfaction.^29^ Inquiring about which symptoms are of the highest priority could enhance the likelihood of a satisfying outcome for both parties in the health care interaction.

Duggan and Street^1^ identified six communication functions key to promoting outcomes: “establishing and maintaining the physician-patient relationship, exchanging and managing information, validating and responding to emotions, managing uncertainty, making treatment decisions, and enabling patient self-management.” Each of these is reflected in the positive codes included in Table 1.

### Negative codes and dissatisfaction with health care interaction

Dissatisfying health care interactions included those in which people receiving care reported invalidating experiences, mismatches in expectations, barriers to treatment, and not feeling helped by the interaction. These themes illustrate the counterpoint to those of satisfying health care interactions and provide lessons for HCPs to consider as they approach midlife patients.

Participants provided several examples of invalidating experiences. Among these were feeling “dismissed,” “brushed off,” or “not feeling heard.” Kennedy et al. found that women attributed changing HCPs to experiences in which communication quality was poor, exemplified by their assertions that the HCP didn't listen, was not concerned or interested, didn't explain enough, didn't spend enough time, or was condescending; they were not heard.^30^

Other invalidating experiences included being told they were “too young” and/or “still having regular cycles” and therefore could not be experiencing symptoms related to the menopausal transition. This resulted in some feeling “blown off.” Then there were situations where the HCP acknowledges that perimenopause may be related to presenting symptoms but advises the person seeking care that they must just accept it. This left some participants feeling that their experiences were trivialized. Yet another response that felt invalidating was perhaps an attempt at humor: “Welcome to menopause!” Although such a comment was likely intended to lighten the conversation, it was not perceived as supportive.

Sitzia and Wood emphasized the role of expectation in patient satisfaction.^31^ Mismatches in expectations underlie many of the negative responses in the WLB survey. Specifically, respondents reported HCPs not endorsing their view of symptoms. These examples resemble those found by Anderson et al. in their qualitative analysis of satisfaction with primary care. Participants reported feeling that their experiences or symptoms were not taken seriously, labeled psychosomatic or attributed to stress, and thus did not feel cared for.^32^ Masse and Legare address the challenges of the health care interaction in perimenopause where the person seeking care's social context has a significant role, as does their view of menopause (*i.e.*, their explanatory model).^33^ The authors emphasize the importance of the HCP eliciting and identifying the patient's view.

Poor communication about how therapies function produced dissatisfaction. Some participants commented about HCPs giving a prescription without clarifying its justification. Others commented that they were offered a drug that they viewed as inappropriate for the problem for which they sought help. This was often a response to being prescribed an antidepressant without an adequate explanation. Antidepressants are an evidence-based therapy for vasomotor symptoms, as well as mood disorders, both common symptoms seen in perimenopause.^34,35^ More people would benefit from knowing this.

Sometimes participants reported that their HCP provided what they thought was incorrect information, or they received conflicting views from different HCPs. These situations may challenge patients' trust in their clinicians, threatening an important relational function in health care.^1^ Green and Ramos conclude that communication, competence, and caring are the key ingredients to build patient trust.^2^ Our qualitative data support this.

Some participants in the WLB survey also identified needing to see multiple HCPs as a barrier to treatment. This was inconvenient, frustrating, and expensive for some. Conversely, some respondents viewed having access to multiple HCPs that worked as team as an asset, as noted above.

A final category of factors associated with dissatisfaction with health care was not feeling helped. HCPs declared “nothing could be done,” performed tests that were uninformative, provided no helpful advice, and/or offered treatment that did not work. Some of these may be instances where the HCP was concerned about ruling out a particular pathologic condition but didn't communicate that effectively to the person receiving care. In other cases, the HCP was unable to offer reassurance about whether perimenopause related symptoms were normal and might attenuate over time or did not have the knowledge to offer management options.

### Opportunities

This analysis highlights several challenges in providing care to midlife, often perimenopausal patients. It also reveals opportunities for improvement of the perimenopausal health care interaction: some at a health care system level and some for the HCPs, themselves. More research is needed about the menopausal transition: when it begins and what symptoms can be attributed to hormonal changes that arise. WLB survey respondents endorsed diverse symptoms all of which manifest before, as well as after, skipped periods.^4^

Our study suggests that validating patient experience, gaining an understanding of the patient's beliefs about their symptoms, and employing shared decision-making are elements of care with potential to promote patient satisfaction. Normalizing the person seeking care's experience as appropriate for age and stage in life, explaining the logic behind a treatment option, or admitting that for some symptoms an exact understanding of cause is not known but that a proposed treatment has been shown to be effective could also improve satisfaction. When a treatment is not available, acknowledging the symptoms while explaining the underlying, sometimes chaotic, hormone fluctuations of perimenopause can be experienced as supportive.

The findings presented here also suggest a lack of knowledge among some HCPs about the wide range of symptoms patients experience and likely are associated with the menopausal transition. A recent survey of trainees in family medicine, obstetrics-gynecology, and internal medicine from several programs corroborates this. Forty percent of these physicians did not feel adequately trained to manage the menopausal transition.^5^ In their article on Women's Health Centers White and Shroff highlight the lack of training in women's health issues, including menopause.^36^ In addition, those in perimenopause are sometimes seen by gynecologists who are not necessarily equipped to handle some common issues such as weight gain, moodiness, and sleep disturbance.^37^

### Limitations

From these data, we could not evaluate how frequently these satisfied and dissatisfied outcomes occur in general midlife care. We did not ask participants what type of provider they sought care from although we know from the open-ended comments that it included primary care, gynecologists, and other specialists. The convenience sample is likely biased toward those with perimenopausal symptoms as the introduction to the survey stated that it was gathering information about either midlife health or the path to menopause. In addition, the symptoms explored in the WLB survey are not always caused by the menopausal transition, although because they are experienced during midlife, they may be attributed to it by either HCRs or midlife patients.

### Future research

Research indicates that patients feel unprepared for the menopausal transition. Alspaugh et al. found that participants in their study received little or no information about menopause from their HCPs.^38^ This was especially disturbing to those who were farther along the path to menopause. Another recent online survey from the United Kingdom revealed that neither those on the path to menopause nor HCPs are adequately educated about menopause.^10^ An evaluation of the impact of education on experiences of the menopausal transition done on a small sample in Turkey suggested that those who were more aware of what to expect had less severe symptoms.^39^ Providing anticipatory guidance and validating/normalizing the menopausal transition deserve further study.

In addition to a better understanding of perimenopausal symptoms and their causes, midlife health care interactions would benefit from further research about which sets of symptoms are amenable to treatment. At present, comparative treatment outcome data to guide care for the array of complaints presented to clinicians during perimenopause are limited, although some has been provided by the MS-FLASH trials.^34,40,41^

It is also important to remember that this major life change, the transition to menopause, has many psychosocial as well as physical implications that are not easily addressed in a short medical visit where clinicians may be prioritizing health maintenance measures and ruling out serious pathology. In their extensive review of the qualitative literature about people's experience of menopause, Hoga et al. conclude that “Healthcare providers pay little attention to women's perceptions regarding menopause.”^42^

In 1997 Mansfield and Voda wrote that “healthcare providers run the risk of invalidating normal menopausal experiences by withholding reassurance that the conditions are commonplace, if annoying or troublesome.”^43^ The desire for education, validation, and support remains an unmet need for those on the path to menopause 25 years later.

## Conclusion

A review of the responses to our open-ended survey question about participants seeking health care for a bothersome midlife symptom demonstrates three opportunities for enhanced care. First, validation is a powerful and underutilized tool in such health care interactions. Second, HCPs who support midlife patients need to know that symptoms on the path to menopause can begin while periods are still regular, that there are a variety of such symptoms (*i.e.*, more than hot flashes and vaginal dryness), and what interaction strategies might lead to more satisfied patients. Finally, anticipatory guidance given before menstrual irregularity about the menopausal transition could improve midlife health care interactions and likely people's experience of this transition. Perhaps it is time to consider a pre-menopause/midlife health visit analogous to a preconception or Medicare wellness visit in which anticipatory guidance is paired with an individualized wellness plan.
